# Perceived Course of Illness on the Desire for Social Distance From People Suffering From Symptoms of Schizophrenia in India

**DOI:** 10.3389/fpsyt.2022.891409

**Published:** 2022-06-03

**Authors:** Gayatri Salunkhe, Kerem Böge, Tanja Wilker, Aron Zieger, Sunita Jena, Aditya Mungee, Thi Minh Tam Ta, Malek Bajbouj, Georg Schomerus, Eric Hahn

**Affiliations:** ^1^Centre of Medicine and Society, Faculty of Medicine, University of Freiburg, Freiburg im Breisgau, Germany; ^2^Department of Psychiatry and Neurosciences, Charité-Universitätsmedizin Berlin, Campus Benjamin Franklin, Berlin, Germany; ^3^Public Health Department, Asian Institute of Public Health, Utkal University, Bhubhaneshwar, India; ^4^Department of Psychiatry, Universitätsklinikum Leipzig, University of Leipzig, Leipzig, Germany

**Keywords:** mental health, stigma and awareness, course of illness, desire for social distance, schizophrenia, India

## Abstract

**Background:**

Stigmatization of people with schizophrenia remains a highly relevant topic worldwide, particularly in low- and middle-income countries like India. It is crucial to identify the determinants of the desire for social distance as a proxy for discriminatory behavior in a socio-cultural context to indicate ways to reduce stigma. This study aims to explore whether the public perception of the perceived course of an illness concerning people with symptoms of schizophrenia has an impact on the desire for social distance.

**Subjects and Methods:**

Data collection took place in five cities in India. The sample (*N* = 447) was stratified for gender, age, and religion. Desire for social distance was sampled based on a self-reported questionnaire using unlabelled vignettes for schizophrenia. First, factor analysis was conducted to identify the main factors underlying the perception of the perceived course of the illness. Subsequently, a regression analysis was conducted to examine the impact of the perception of those prognostic factors on the desire for social distance.

**Results:**

Factor analysis revealed two independent factors of the perceived course of an illness: (1) *life-long dependency on others and loss of social integration and functioning* and (2) *positive expectations toward treatment outcome*. This second factor was significantly associated with a less desire for social distance toward persons with schizophrenia.

**Conclusion:**

The desire for social distance toward people with schizophrenia reduces with the expectation of positive treatment outcomes which underlines the need to raise public mental health awareness and provide psychoeducation for affected people and their family members in India. Help-seeking behaviors can be promoted by directing those needing treatment toward locally available, affordable and credible community-based services rather than facility-based care. Strikingly, lifelong dependency and the inability to socially integrate do not increase the desire for social distance, reflecting the Indian nation's socio-relational values and insufficiency of public mental health services. This indicates the suitability of systemic therapy approaches in public mental healthcare services to support the family's involvement and family-based interventions in caregiving for mentally ill people across the lifespan.

## Introduction

India's population of 1.4 billion inhabitants is currently the second-largest globally and is predicted to become the largest by 2023 (1). At the same time, India belongs to lower and middle-income countries (LMICs) (2), which poses significant challenges to delivering healthcare and providing universal health coverage for the whole country. With a mental health treatment gap as high as 83% (3), mental health issues are one of the leading causes of years lived with disability (YLD) in India (4). Yet, <1% of India's health budget is spent on mental healthcare, translating to only 0.003 psychiatric hospitals (5) and 0.3 psychiatrists per 100,000 people in India (6). Furthermore, most mental health facilities and specialists are concentrated in Indian metropolitan cities, while access to care in rural areas is a major issue. Evidence suggests a negative cycle between mental illness and poverty in LMICs like India. Still, most mental health services are not covered as part of universal health coverage and are typically out-of-pocket expenses (7).

Schizophrenia is a severe mental disorder and is among the leading contributors to the global disease burden (8). A major challenge for persons with severe mental disorders like schizophrenia is providing services in line with human rights (9). After ratifying the Convention on Rights of Persons with Disabilities (CRPD) established by the United Nations (UN) (10) in 2007, India developed and established two important enactments to comply with the CRPD: The Mental Health Care Act 2017 (MHCA) (11) and the Rights of Persons with Disability Act 2016 (RPDA) (12). These Acts represent a necessary step toward credible mental health care provision in India. They clearly articulate and thereby broaden the rights for mental health care and treatment of persons with mental illness through a rights-based approach. However, diverse conceptual and practical challenges remain in properly implementing these Acts due to complex shortcomings in the nation's mental health care system as well as stigmatization and discrimination toward affected persons and their caregivers.

Another barrier to mental health service utilization in India is stigma toward and discrimination against people with severe mental disorders (13), particularly for psychotic disorders such as schizophrenia (14). The concept of stigma is related to a lack of knowledge, negative attitudes and discrimination by excluding persons or avoiding persons with mental illness (15). Stigma is a devaluating relationship in which one person is “disqualified from full social acceptance” (16). As the definition emphasizes, stigmatization is largely socially constructed. As different societies develop different norms, which in turn shape behaviors, beliefs, standards, and values, stigmatization needs to be understood in its local contexts so that programs can be developed accordingly and fundamentally change attitudes toward people with mental illnesses in the long term.

Schizophrenia is one of the most stigmatized disorders due to the perception of dangerousness and aggressiveness initiated by affected persons (17). Those preconceptions, in turn, can lead to a sense of shame, social isolation, increased mental distress and even an increased risk for suicidal ideation for affected individuals (18). Furthermore, in India, the stigmatization of people with schizophrenia affects their social surroundings, including hindered access to work and reduced ability to form stable relationships, thereby fostering neglect and marginalization (19, 20). It is paramount to reduce stigma to increase well-being as well as the chances for better chances of adequate recovery for people who have a mental disorder.

In India, stigma-related preoccupations primarily concern specific expectations of gender and societal roles as well as a social line of conduct (21). The most noteworthy stigma-related hindrances of people who have a mental illness are reduced chances for employment, being part of the community, and fears about neighbors avoiding them or treating them differently (19, 20). Studies have shown that those preoccupations affect people with schizophrenia, their family members and caregivers, which consecutively lead to feelings of depression or helplessness. Accordingly, caregivers regularly feel uncomfortable disclosing their family member's condition, resulting in a high level of estrangement, stereotype endorsement and discrimination experience in India (19–21). Stigma can also impact and delay help-seeking behaviors. According to an Indian study for persons with schizophrenia, stigma is positively correlated with seeking informal care and negatively associated with seeking allopathic care (22).

The desire for social distance can be considered a measurable proxy for discriminatory behavior and is a crucial variable when investigating stigmatization and discrimination (23). Public assumptions and expectations *concerning an illness* are closely linked to their reactions *toward the ill* (24). Modifying false beliefs or expectations can thus be one of the central mechanisms for reducing stigma (25). In an Indian sample of metropolitan participants, the present study analyses how the expectations about the course of mental illness in individuals showing symptoms of schizophrenia are related to desire for social distance.

## Methods

### Participants

Data collection took place between April and July 2014. The accurate and consistent completion of the questionnaires was ensured through a trained market research provider Panoramix^TM^. Since the participants who took part in the survey belonged to a pool registered with the market research firm, the study was carried out using a convenience sample. The *inclusion criteria* were defined as participants between the age group of 18 to 65 years who agreed to be contacted by the research firm beforehand and consented to participate on an informed basis. Data collection was first initially performed in Chennai and Kolkata, whereby the samples of these two cities were matched. Subsequently, data were collected from three different cities through quota sampling of gender, age, and religion using data from participants from Chennai and Kolkata. The current sample is part of a sample from a larger study (21). In line with the scope of this study, only participants who were answering vignette-based questionnaires describing symptoms of schizophrenia were included. This resulted in a convenience sample of 447 participants from 5 metropolitan Indian cities (Chennai = 69, Kolkata = 73, Hyderabad = 64, Mumbai = 148, and Lucknow = 93). Detailed demographic characteristics of the current study sample and comparisons with the 2011 census data (26) are presented in Table 1.

**Table 1:** Sociodemographic characteristics of survey sample in percentage in comparison to the total population of India according to the census of 2011.

**Sociodemographic data**	**2011 Census data (*N* = 1,210,854,977) %**	**Schizophrenia vignette sample (*N* = 447)**
Gender (%)		
Male	51.47	52.8
Female	48.53	47.2
Age, years (%)		
18–24	30.21	20.4
25–34	24.75	35.3
35–44	20.53	29.5
45–54	14.51	13
55–64	10	1.8
School education (%)		
Primary School or lower	45.64	4.9
Middle School	18.46	13.9
Secondary or higher	35.91	81.2
Strong religious beliefs (%)		
Yes	N/A	69.1
No	N/A	30.9
Religion (%)		
Hinduism	79.80	80.1
Islam	14.23	9.8
Christianity	2.30	4.7
Other	3.67	5.4

*All data are given in percentages. Data from India census 2011. N/A, Not available*.

### Questionnaires

The study was conducted with vignette-based questionnaires, which have been used in prior Western (27–29) and Indian studies (30). Vignettes described a person with symptoms defining schizophrenia according to ICD-10 (31) as well as DSM-IV (32) criteria. According to the recommended guidelines, the English questionnaires were translated into the local languages spoken in the five cities and responses were translated back into English (33). All versions were checked for consistency and semantics by two independent certified translators. The interviews were conducted based on a fully-structured questionnaire by psychologists who used a combination of guided self-report and interview methods, depending on the literacy and preferences of the respondents. The questionnaire took about 25 min to complete.

### Perceptions of the Course of Illness

To assess the participant's perception of the course of illness, respondents were asked to rate their agreement toward eight statements on the perceived course of illness using a five-point Likert scale. Answer possibilities ranged from (1) “Definitely true” to (5) “Definitely not true”. Participants stated whether they thought that the person would fully recover, would ever be able to make important decisions by themselves again, neglected themselves, would be able to live a normal life again, would always be in need of help from others, would have a regular job, would always need someone to tell them what to do or whether their condition would significantly improve, and if treatment would help.

### Desire for Social Distance

For assessing desire for social distance, the well-established Social Distance Scale (SDS) developed and described by Link et al. was used (23, 27–30). This scale is comprised of eight items, whereby respondents rate their opinion on a five-point Likert scale. Answer possibilities ranged from (1) “I would definitely” to (5) “I most definitely would not”. The given contexts include whether they would accept the individual as a subtenant, a colleague at work, a neighbor, a person who takes care of their children, a family member in-law, if they would introduce this person to a friend or if they would recommend them for a job.

### Statistical Analysis

Statistical analysis was performed using the IBM program SPSS Statistics for Mac OS X Version 21. First, two of the eight statements on the perception of the course of illness were reverse-coded, as they were framed positively during the interview (“after treatment, he/she will be able to live a normal life again” and “with treatment his/her condition will significantly improve”) whereas the other statements were framed negatively (for example “He/She will never be able to have a regular job”). Subsequently, using principal factor extraction and varimax rotation, a factor analysis including the eight items was conducted to define the underlying unifying factors concerning the expectations for the course of illness. Only factors with an eigenvalue greater than Kaiser's criterion of one were extracted. The Kaiser-Meyer-Olkin measure of sampling adequacy (KMO) resulted in a value of 0.808 (*p* < 0.001), rendering the sample acceptable for factor analysis. Individual factor scores for each participant were saved as Anderson-Rubin variables. For the desire for social distance, a sum score for each participant was calculated by adding together the numbers of the corresponding responses. Next, a regression analysis was carried out using the social distance sum scores as dependent variables and the individual Anderson-Rubin variables as independent variables. An association of factors with desire for social distance was set for *p* < 0.05.

## Results

### Socio-Demographic Characteristics

The socio-demographic information summarized in Table 1 shows that our sample was predominantly Hindu by religion and consisted of a slightly larger proportion of males, overall comparable with the 2011 Indian census data. A majority of participants in our sample were between 35 and 44 years of age where as a majority of the population was between 18 and 24 years of age according to the 2011 census data. Ours was a highly educated sample, with a majority of participants having Secondary education or higher, while the majority of the population according to the 2011 census data had Primary education or lower.

### Factor Extraction for the Perception of the Course of Illness

For the factor analysis, eight items concerning the perceived course of illness for the person depicted in the vignette as suffering from symptoms of schizophrenia were included. Two independent underlying factors had an eigenvalue greater than Kaiser's criterion of one and could therefore be extracted (see Table 2): first, (1) *loss of social functioning and lifelong dependency on others* comprised of the items “the person will never fully recover, “the person will never be able to make important decisions by him or herself”, “the person will neglect him/herself”,” the person will never be able to have a regular job”, “the person will always be in need of help from others”, and “the person will always need someone to tell him/her what to do”. Second, (2) *positive expectations toward treatment outcome* involving the items “after treatment, the person will be able to live a normal life again” and “with treatment, the person's condition will significantly improve.”

**Table 2:** Summary of exploratory factor analysis results for illness course perception.

**Rotated factor loadings**		
**Item**	**1**	**2**
The person will never be able to make important decisions by him/herself	**0.838**	0.098
The person will never be able to have a regular job	**0.810**	0.110
The person will never fully recover	**0.786**	0.244
The person will always need someone to tell him/her what to do	**0.769**	−0.097
The person neglects his/herself	**0.701**	0.052
The person will always be in need of help from others	**0.694**	−0.150
With treatment, the person's condition will significantly improve (reverse coded)	0.048	**0.853**
After treatment, the person will be able to live a normal life again (reverse coded)	0.019	**0.835**
Initial Eigenvalues	3.581	1.502
% of variance	44.767	18.771

*The table shows the two extracted factors after principal factor extraction and varimax rotation with their initial eigenvalues and the percentage of variance explained. Items: 1. Loss of social functioning and lifelong dependency on others, 2. Positive expectations toward treatment outcome*.

### Regression Analysis for the Perception of the Course of Illness on Social Distance

A linear regression analysis has been performed to determine the impact of the extracted factors underlying the perception of the course of illness on the desire for social distance. It showed that one of the two factors significantly affected the desire for social distance with *p* < 0.01, namely the second factor (2) *positive expectations toward treatment outcome*. The factor (1) *loss of social functioning and lifelong dependency on others* had no significant effect on our sample's desire for social distance (*p* = 0.19). Table 3 shows a summary of the regression analysis.

**Table 3:** Regression analysis for perception of course of illness as independent and desire for social distance as a dependent variable.

	***b***	**β**	***p***
Constant	18.729		0.000*
(1) Loss of social functioning and lifelong dependency on others	−0.348	−0.061	0.190
(2) Positive expectations toward treatment outcome	−1.175	−0.206	0.000*

The table shows the results of linear regression analysis of predictors of the desire for social distance with beta scores, standardized beta values, and their significance value. Significant predictors are in bold and marked with * for p < 0.01.

## Discussion

The study at hand investigated whether the perception of the course of an illness influenced the public's desire to social distance themselves from patients suffering from symptoms of schizophrenia in India. We found, *firstly*, that the desire for social distance toward persons with schizophrenia is lowered if expectations toward treatment outcomes are positive, but *secondly*, that it is unaffected by lifelong dependency on others and loss of social integration and functioning. The interpretations and implications of these findings are discussed below.

The main finding of this study was that positive perceptions and attitudes concerning the course of illness correlated with reduced desire for social distance in India, replicating findings of previous well-established studies in different countries worldwide, independent of their economic developmental state (34, 35). In Germany as well as Tunisia, the expectation of a person suffering from symptoms of schizophrenia regaining a healthy status leads to more pro-social activities and, consequently, less desire for social distance (36). Furthermore, Martensen et al. (34) reported the same significant relationship between favorable perceptions of the course of an illness with lowered desire for social distance from a sample of the metropolitan region in Vietnam. This finding underlines the importance of spreading awareness at a societal level and providing psychoeducation to family members about schizophrenia and positive treatment outcomes of comprehensive bio-psycho-social interventions (37) to reduce desire for social distance from persons with schizophrenia in India.

Commendable efforts have been made in recent years to raise public mental health awareness by Indian governmental and non-governmental organizations, celebrities and mental health professionals, yet a lot more has to be implemented (38). While these initiatives are likely to increase help-seeking behaviors, they have been criticized for lacking qualitative value in communicating to the public from *whom* help can be sought (38). A majority of Indians would deem psychiatrists the preferred professional to consult for severe mental health issues due to the perception of being more knowledgeable, easier to find, and quickly prescribed medication (38). However, this perception is problematic since India has only 0.75 psychiatrists per 100,000 persons against a standard reference of at least 3 psychiatrists per 100,000 persons (6).

Efforts to raise the acceptability of seeking mental health care are only ethical if the required services are given. In LMICs, most persons with schizophrenia do not receive adequate treatment (39), and if sought, the treatment is primarily hospital-based, led by specialists with little attention to non-pharmacological aspects. Fortunately, evidence suggests that the challenges of availability, accessibility, affordability, acceptability and credibility of mental health services for schizophrenia in India can feasibly be addressed by employing collaborative community-based care rather than facility-based care (40–43). The community-based care model involves scaling-up interventions through task-sharing, whereby locally available community healthcare workers (CHWs) are trained to provide the largest share of service delivery and work in collaboration with health specialists, persons with schizophrenia, and their family members well as local social welfare organizations. According to this model (44), CHWs are responsible for spreading awareness, early detection of new and relapse cases, referral to specialists, providing low-intensity psychosocial support to affected persons and their family members, and promoting social inclusion. Mental health specialists are in charge of diagnoses, drug and specialized psychosocial treatment, supervising CHWs and providing in-patient care. The development of such collaborative community-based services for mental health illnesses in LMICs has become a global health priority (44); it is thus astute that the awareness about schizophrenia and help-seeking behaviors is aligned accordingly. Given the lack of mental health specialists in India (6), it is suitable for future initiatives to inform the public about the collaborative nature of mental health services, the role of CHWs as gate-keepers in the health care system, and where and how they can be reached.

Since most persons with schizophrenia live with their families in India and rely on them for financial support and decisions related to treatment (45–48), these caregivers must receive sufficient support to help understand the illness and help them cope with the demanding caregiving process. Psychoeducation of persons and family members is important as self-stigma, the internalization of negative attitudes, and family shame are predictors of treatment avoidance (49). Reports showed that psycho-educational interventions reduced psychopathology and disability for persons with schizophrenia and increased support and satisfaction for their caregivers in India (50) and that the delivery of such interventions by CHWs is feasible and acceptable in the Indian context (43). An example of cultural adaptation was considering the delivery of interventions in community spaces rather than at home to reduce fear of illness disclosure and subsequent social exclusion by the community (43). In terms of the content of these psycho-educative interventions in India, it is recommended that rather than focusing on the biomedical explanations for schizophrenia, an emphasis should be placed on the possibility for recovery and fulfilled lives if appropriate treatment is sought using messages like “recovery is possible” and “no-one is to blame” (20).

Our second finding was that *loss of social functioning and lifelong dependency on others* did not increase the desire for social distance within the Indian context, contradicting prior findings in Vietnam (34). A potential reason for the inconsistency in these findings is that stigmatization is a cultural-sensitive phenomenon and should be considered in the light of different cultural habits and norms (51). A comparison between Vietnam and India seems especially interesting, as both countries represent LMICs that are transitioning into middle-income countries. Nevertheless, both cultures are highly dissimilar, as the values of Vietnamese people are influenced by different beliefs and traditions, among them social-relational concepts leaning on Confucianism and expectations of reciprocity (52, 53). A lifelong dependency on others may reflect a perceived failure of expected reciprocity behaviors on the dependants and, in turn, explain an increased desire for social distance. When viewing western cultures, individualism is valued, emphasizing personal independence, autonomy, individual recognition and personal success (54). Here, people with mental illnesses can be perceived as unable to achieve individual goals and needing assistance from others, which can contribute to their perceived or experienced stigmatization (54).

Despite their heterogeneity, Asian, African and Latino cultures share the common strands of collectivism and interdependence (55). This is also true for the Indian society, where family integrity, loyalty and unity, family members and extended kin are traditionally involved in the treatment process of the family member affected by a mental illness (47, 48). They are also involved in decision-making related to employment and marriage (45), topics that are highly relevant during early adulthood when schizophrenia typically onsets. The benefits of family involvement in the Indian context are evident as individuals with schizophrenia are more socially integrated (56, 57) and have a more stable course for the illness than their western counterparts (58, 59).

In addition to socio-relational values, family involvement may be influenced by the structural and financial situation of the public mental healthcare system. For example, Canadians assign more responsibility to their government concerning mental health care needs than Indians, whereas Indians assign more responsibility to families than do Canadians (60). The stark differences between the public healthcare systems need to be acknowledged here, with universal healthcare benefits bearing around 70% of healthcare costs through public funding (61) and subsidized housing for individuals with mental illness in Canada (60). At the same time, India struggles with extreme inadequacy of mental health services, with 82% of healthcare expenses being covered out-of-pocket and virtually no government-supported or subsidized housing (62). Consequently, lifelong dependency on others having no effect on the desired social distance in India can be understood as a traditional way of living whereby family members feel an intense emotional interdependence and loyalty to each other in a context of insufficient public mental healthcare services. To protect and empower the Indian family support system, an essential resource in low-resource settings, family members must receive psychosocial support at the grassroots level *via* trained CHWs (43), self-help groups and governmental policies in favor of social benefits (63).

The present study should be seen in the light of various limitations. India is a highly diverse country, composed of multiple culturally diverse communities. Therefore, the urban population from 5 large Indian cities in different regions of this vast and diverse country that participated in this study cannot be generalized to the whole country, especially to more rural areas. The educational level of our sample was higher than the average Indian population and the Census 2011. As former studies established, lower education is correlated with higher stigmatization of the psychiatric field (16). Consequently, stigmatization in our sample could be lower due to the heightened educational level of the participants. Previous studies reported that perceived stigma toward people with mental illness was higher amongst Indian females than Indian males, which may be due to gender and societal roles (21). The influence of socio-demographic factors like education levels, gender, income levels, religious background, family size and familiarity with mental illness is unclear and should be explored in future research. While the SDS is a well-established instrument that has previously been validated in Indian samples, ours was the first study to administer the questionnaire on *perceptions of course of illness* in India. It should also be considered that face-to-face interviews could have led people not to answer according to perceived expectations or be led to a specific answer pattern by the way the interviewer asked the questions. Additionally, face-to-face interviews are prone to perceived social desirability while answering. Moreover, since the study design was cross-sectional and the results can only display mere correlations, no implications of causality can be made.

## Conclusion

Stigma toward persons with schizophrenia can potentially be reduced if the Indian society is informed about the favorable prognosis for this illness when appropriate treatment is provided. Awareness and psychoeducation programs should encourage help-seeking behaviors and inform affected people and their family members where and from whom care can be sought. Simultaneously, the demand for and supply of affordable, accessible and credible community-based services over specialized facility-based services needs to be boosted. Here, providing psychosocial services for the extended family is a must as their involvement based on values of interdependence and inclusion are critical to the caregiving process within the Indian context.

## Data Availability Statement

The raw data supporting the conclusions of this article will be made available by the authors, without undue reservation.

## Ethics Statement

The studies involving human participants were reviewed and approved by Asian Institute of Public Health, Bhubaneshwar, India. Verbal informed consent was obtained from each participant before the interview. Respondents were made aware of the research objective and purpose of the study. Participation in this study was completely voluntary. All data was saved in an anonymized statistical form. Written informed consent for participation was not required for this study in accordance with the national legislation and the institutional requirements.

## Author Contributions

The writing of the first draft manuscript was led by GSa and all following versions were supported by KB, TW, AZ, SJ, AM, TT, MB, GSc, and EH. Statistical analyses were conducted by KB and TW. The study design was prepared by TT, AM, AZ, and EH. Data collection was supervised by AZ, AM, and EH. All authors contributed to the article and approved the submitted version.

## Conflict of Interest

The authors declare that the research was conducted in the absence of any commercial or financial relationships that could be construed as a potential conflict of interest.

## Publisher's Note

All claims expressed in this article are solely those of the authors and do not necessarily represent those of their affiliated organizations, or those of the publisher, the editors and the reviewers. Any product that may be evaluated in this article, or claim that may be made by its manufacturer, is not guaranteed or endorsed by the publisher.

## References

[1] United Nations Department of Economic Social Affairs Population Division. World Population Prospects: The 2015 Revision, Volume I: Comprehensive Tables (ST/ESA/SER.A/379). (2015). Available online at: https://population.un.org/wpp/Publications/Files/WPP2015_Volume-I_Comprehensive-Tables.pdf (accessed April 30, 2022).

[2] The World Bank. World Bank Country and Lending Groups. Available online at: https://datahelpdesk.worldbank.org/knowledgebase/articles/906519 (accessed April 30, 2022).

[3] Gururaj G Varghese M Benegal V Rao GN Pathak K Singh LK. NMHS collaborators group. National mental health survey of India. (2015) 16:30–2.

[4] Sagar R Dandona R Gururaj G Dhaliwal RS Singh A Ferrari A. The burden of mental disorders across the states of India: the Global Burden of Disease Study 1990–2017. Lancet Psychiat. (2020) 7:148–61. 10.1016/S2215-0366(19)30475-4 31879245 PMC7029418

[5] Patel V Xiao S Chen H Hanna F Jotheeswaran AT Luo D. The magnitude of and health system responses to the mental health treatment gap in adults in India and China. Lancet. (2016) 388:3074–84. 10.1016/S0140-6736(16)00160-4 27209149

[6] Garg K Kumar CN Chandra PS. Number of psychiatrists in India: Baby steps forward, but a long way to go. Indian J Psychiatry. (2019) 61:104. 10.4103/psychiatry.IndianJPsychiatry_7_18 30745666 PMC6341936

[7] Lund C Breen A Flisher AJ Kakuma R Corrigall J Joska JA. Poverty and common mental disorders in low and middle-income countries: a systematic review. Soc Sci Med. (2010) 71:517–28. 10.1016/j.socscimed.2010.04.027 20621748 PMC4991761

[8] Charlson FJ Ferrari AJ Santomauro DF Diminic S Stockings E Scott JG. Global epidemiology and burden of schizophrenia: findings from the global burden of disease study 2016. Schizophr Bull. (2018) 44:1195–203. 10.1093/schbul/sby058 29762765 PMC6192504

[9] Patel V Saxena S Lund C Thornicroft G Baingana F Bolton P. The Lancet Commission on global mental health and sustainable development. Lancet. (2018) 392:1553–98. 10.1016/S0140-6736(18)31612-X 30314863

[10] United Nations and Convention on the rights of persons with disabilities. New York: United Nations (2006).

[11] Ministry of Law and Justice. The Metal Health Care Act (2017).

[12] The Rights of Persons with Disabilities Act. The Gazette of India (2016).

[13] Shidhaye R Kermode M. Stigma and discrimination as a barrier to mental health service utilization in India. Int Health. (2013) 5:6–8. 10.1093/inthealth/ihs011 24029838

[14] Grover S Avasthi A Singh A Dan A Neogi R Kaur D. Stigma experienced by patients with severe mental disorders: A nationwide multicentric study from India. Psychiatry Res. (2017) 257:550–8. 10.1016/j.psychres.2017.08.027 28918241

[15] Thornicroft G Rose D Kassam A Sartorius N. Stigma: ignorance, prejudice or discrimination? Brit J Psychiat. (2007) 190:192–3. 10.1192/bjp.bp.106.025791 17329736

[16] Thara R Srinivasan TN. How stigmatising is schizophrenia in India? Int J Soc Psychiatry. (2000) 46:135–41. 10.1177/002076400004600206 10950361

[17] Taskin EO Seyfe Sen F Aydemir O Demet MM Ozmen E Icelli I. Public attitudes to schizophrenia in rural Turkey. Soc Psychiatry Psychiatr Epidemiol. (2003) 38:586–92. 10.1007/s00127-003-0655-y 14564386

[18] Sharaf AY Ossman LH Lachine OA. A. cross-sectional study of the relationships between illness insight, internalized stigma, and suicide risk in individuals with schizophrenia. Int J Nurs Stud. (2012) 49:1512–20. 10.1016/j.ijnurstu.2012.08.006 22939218

[19] Koschorke M Padmavati R Kumar S Cohen A Weiss HA Chatterjee S. Experiences of stigma and discrimination of people with schizophrenia in India. Soc Sci Med. (2014) 123:149–59. 10.1016/j.socscimed.2014.10.035 25462616 PMC4259492

[20] Koschorke M Padmavati R Kumar S Cohen A Weiss HA Chatterjee S. Experiences of stigma and discrimination faced by family caregivers of people with schizophrenia in India. Soc Sci Med. (2017) 178:66–77. 10.1016/j.socscimed.2017.01.061 28213300 PMC5360174

[21] Böge K Zieger A Mungee A Tandon A Fuchs LM Schomerus G. Perceived stigmatization and discrimination of people with mental illness: A survey-based study of the general population in five metropolitan cities in India. Indian J Psychiatry. (2018) 60:24. 10.4103/psychiatry.IndianJPsychiatry_406_17 29736059 PMC5914258

[22] Raguram R Raghu TM Vounatsou P Weiss MG. Schizophrenia and the cultural epidemiology of stigma in Bangalore, India. J Nerv Ment Dis. (2004) 192:734–44. 10.1097/01.nmd.0000144692.24993.1b 15505517

[23] Corrigan PW Edwards AB Green A Diwan SL Penn DL. Prejudice, social distance, and familiarity with mental illness. Schizophr Bull. (2001) 27:219–25. 10.1093/oxfordjournals.schbul.a006868 11354589

[24] Schwarz KA Pfister R Büchel C. Rethinking explicit expectations: connecting placebos, social cognition, contextual perception. Trends Cogn Sci. (2016) 20:469–80. 10.1016/j.tics.2016.04.001 27108268

[25] Rief W Glombiewski JA. The role of expectations in mental disorders and their treatment. World Psychiatry. (2017) 16:210. 10.1002/wps.20427 28498580 PMC5428191

[26] Census of India. New Delhi: Office of Registrar General and Census Commissioner, India (2011). Available online at: http://censusindia.gov.in/2011-Common/CensusData2011.html (accessed April 30, 2022).

[27] Link BG Cullen FT Frank J Wozniak JF. The social rejection of former mental patients: understanding why labels matter. Am J Sociol. (1987) 92:1461–500. 10.1086/228672

[28] Schomerus G Matschinger H Angermeyer MC. Continuum beliefs and stigmatizing attitudes towards persons with schizophrenia, depression and alcohol dependence. Psychiatry Res. (2013) 209:665–9. 10.1016/j.psychres.2013.02.006 23465293

[29] Angermeyer M Daubmann A Wegscheider K Mnich E Schomerus G Knesebeck O. The relationship between biogenetic attributions and desire for social distance from persons with schizophrenia and major depression revisited. Epidemiol Psychiatr Sci. (2015) 24:335–41. 10.1017/S2045796014000262 24786227 PMC7192186

[30] Mathias K Kermode M Goicolea I Seefeldt L Shidhaye R San Sebastian M. Social distance and community attitudes towards people with psycho-social disabilities in Uttarakhand, India. Community Ment Health J. (2018) 54:343–53. 10.1007/s10597-017-0211-y 29143156

[31] Dilling H. Internationale Klassifikation psychischer Störungen (2015).

[32] American Psychiatric Association. Diagnostic and Statistical Manual of Mental Disorders. 4th ed. Washington, DC: American Psychiatric Association (1994).

[33] Beaton DE Bombardier C Guillemin F Ferraz MB. Guidelines for the process of cross-cultural adaptation of self-report measures. Spine. (2000) 25:3186–91. 10.1097/00007632-200012150-00014 11124735

[34] Martensen LK Hahn E Cao TD Schomerus G Nguyen MH Böge K. Impact of perceived course of illness on the desire for social distance towards people with symptoms of schizophrenia in Hanoi, Vietnam. Psychiatry Res. (2018) 268:206–10. 10.1016/j.psychres.2018.05.046 30055410

[35] Lauber C Nordt C Falcato L Rössler W. Factors influencing social distance toward people with mental illness. Community Ment Health J. (2004) 40:265–74. 10.1023/B:COMH.0000026999.87728.2d 15259631

[36] Angermeyer MC Carta MG Matschinger H Millier A Refai T Schomerus G. Cultural differences in stigma surrounding schizophrenia: comparison between Central Europe and North Africa. Brit J Psychiat. (2016) 208:389–97. 10.1192/bjp.bp.114.154260 26585098

[37] World Health Organization. Comprehensive Mental Health Action Plan 2013–2030. Geneva: World Health Organization (2021).

[38] Saha S. The glitch in mental health awareness campaigns in India. J Psychosoc Res. (2018) 13:131–9. 10.32381/JPR.2018.13.01.14

[39] Lora A Kohn R Levav I McBain R Morris J Saxena S. Service availability and utilization and treatment gap for schizophrenic disorders: a survey in 50 low- and middle-income countries. Bull World Health Organ. (2012) 90:47–54. 10.2471/BLT.11.089284 22271964 PMC3260570

[40] Chatterjee S Leese M Koschorke M McCrone P Naik S John S. Collaborative community-based care for people and their families living with schizophrenia in India: protocol for a randomised controlled trial. Trials. (2011) 12:1–14. 10.1186/1745-6215-12-12 21226970 PMC3033834

[41] Chatterjee S Pillai A Jain S Cohen A Patel V. Outcomes of people with psychotic disorders in a community-based rehabilitation programme in rural India. Brit J Psychiat. (2009) 195:433–9. 10.1192/bjp.bp.108.057596 19880934 PMC2806571

[42] Chatterjee S Naik S John S Dabholkar H Balaji M Koschorke M. Effectiveness of a community-based intervention for people with schizophrenia and their caregivers in India (COPSI): a randomised controlled trial. Lancet. (2014) 383:1385–94. 10.1016/S0140-6736(13)62629-X 24612754 PMC4255067

[43] Balaji M Chatterjee S Koschorke M Rangaswamy T Chavan A Dabholkar H. The development of a lay health worker delivered collaborative community-based intervention for people with schizophrenia in India. BMC Health Serv Res. (2012) 12:1–12. 10.1186/1472-6963-12-42 22340662 PMC3312863

[44] Patel V. Universal health coverage for schizophrenia: a global mental health priority. Schizophr Bull. (2016) 42:885–90. 10.1093/schbul/sbv107 26245942 PMC4903041

[45] Chadda RK Deb KS. Indian family systems, collectivistic society and psychotherapy. Indian J Psychiatry. (2013) 55:S299. 10.4103/0019-5545.105555 23858272 PMC3705700

[46] Avasthi A. Preserve and strengthen family to promote mental health. Indian J Psychiatry. (2010) 52:113–26. 10.4103/0019-5545.64582 20838498 PMC2927880

[47] Thara R Padmavati R Srinivasan TN. Focus on psychiatry in India. Brit J Psychiat. (2004) 184:366–73. 10.1192/bjp.184.4.366 15104094

[48] Mullaiti L. Families in India: Beliefs and realities. J Comp Fam Stud. (1995) 26:11–25. 10.3138/jcfs.26.1.11

[49] Corrigan P. How stigma interferes with mental health care. Am Psychol. (2004) 59:614–25. 10.1037/0003-066X.59.7.614 15491256

[50] Kulhara P Chakrabarti S Avasthi A Sharma A Sharma S. Psychoeducational intervention for caregivers of Indian patients with schizophrenia: a randomised-controlled trial. Acta Psychiatr Scand. (2009) 119:472–83. 10.1111/j.1600-0447.2008.01304.x 19032700

[51] Kermode M Bowen K Arole S Pathare S Jorm AF. Attitudes to people with mental disorders: a mental health literacy survey in a rural area of Maharashtra, India. Soc Psychiatry Psychiatr Epidemiol. (2009) 44:1087–96. 10.1007/s00127-009-0031-7 19305937

[52] Nguyen QTN. The vietnamese values system: a blend of oriental, western and socialist values. Int Educ Stud. (2016) 9:32–40. 10.5539/ies.v9n12p32

[53] Yum JO. The impact of Confucianism on interpersonal relationships and communication patterns in East Asia. Commun Monogr. (1988) 55:374–88. 10.1080/03637758809376178

[54] Mizelle ND. Counseling white americans. In: Marini I, Stebnicki MA, editors. The Professional Counselor's Desk Reference. New York, NY: Springer (2009). p. 247–54.

[55] Abdullah T Brown TL. Mental illness stigma and ethnocultural beliefs, values, and norms: an integrative review. Clin Psychol Rev. (2011) 31:934–48. 10.1016/j.cpr.2011.05.003 21683671

[56] Sharma V Murthy S Agarwal M Wilkinson G. Comparison of people with schizophrenia from Liverpool, England and Sakalwara-Bangalore, India. Int J Soc Psychiatry. (1998) 44:225–30. 10.1177/002076409804400308

[57] Kulhara P Chakrabarti S. Culture and schizophrenia and other psychotic disorders. Psychiatr Clin N Am. (2001) 24:449–64. 10.1016/S0193-953X(05)70240-9 11593856

[58] Thara R Henrietta M Joseph A Rajkumar S Eaton WW. Ten-year course of schizophrenia—the Madras longitudinal study. Acta Psychiatr Scand. (1994) 90:329–36. 10.1111/j.1600-0447.1994.tb01602.x 7872036

[59] Eaton WW Thara R Federman B Melton B Liang KY. Structure and course of positive and negative symptoms in schizophrenia. Arch Gen Psychiatry. (1995) 52:127–34. 10.1001/archpsyc.1995.03950140045005 7848048

[60] Iyer SN Malla A Pope M Mustafa S Mohan G Rangaswamy T. Whose responsibility? Part 2 of 2: views of patients, families, and clinicians about responsibilities for addressing the needs of persons with mental health problems in Chennai, India and Montreal, Canada. Int J Ment Health Syst. (2022) 16:1–16. 10.1186/s13033-021-00511-w 35000588 PMC8744303

[61] Martin D Miller AP Quesnel-Vallée A Caron NR Vissandjée B Marchildon GP. Canada's universal health-care system: achieving its potential. Lancet. (2018) 391:1718–35. 10.1016/S0140-6736(18)30181-8 29483027 PMC7138369

[62] Gopalrao Swaminath AE Rao R Kumar KVK Kumar CN. Mental Healthcare Act, 2017 and homeless persons with mental illness in India. Indian J Psychiatry. (2019) 61:S768. 10.4103/psychiatry.IndianJPsychiatry_117_19 31040471 PMC6482680

[63] Seshadri K Sivakumar T Jagannathan A. The Family Support Movement and Schizophrenia in India. Curr Psychiatry Rep. (2019) 21:1–7. 10.1007/s11920-019-1081-5 31522258

